# Telocytes Twenty Years on: A Critical Reappraisal of Identity, Function, and Pathological Relevance

**DOI:** 10.3390/ijms27146204

**Published:** 2026-07-11

**Authors:** Luciana Alexandra Pavelescu, Sanda Maria Crețoiu

**Affiliations:** Department of Morphological Sciences, Cell and Molecular Biology and Histology Discipline, Carol Davila University of Medicine and Pharmacy, 050474 Bucharest, Romania; luciana.pavelescu@umfcd.ro

**Keywords:** telocytes, telopodes, stromal network organiser, telocyte-associated stromal network dysfunction, Wnt signalling, fibrosis, extracellular vesicles, stem-cell niche, evidence matrix

## Abstract

Telocytes are stromal cells defined by extremely long, moniliform prolongations termed telopodes and have been described in most mammalian organs since their formal designation in 2010. During the past two decades, the field has expanded from ultrastructural organ-mapping to hypotheses concerning stem-cell niche regulation, extracellular-vesicle-mediated communication, fibrosis, inflammation, and cancer-associated stromal remodelling. This expansion, however, has also generated methodological heterogeneity, with frequent reliance on non-specific markers such as CD34, PDGFRα, vimentin, and c-kit, often without ultrastructural validation or functional perturbation. In this critical review, we reassess the evidential status of major claims in telocyte biology, distinguishing robustly demonstrated mechanisms from plausible but incompletely proven hypotheses. The strongest functional evidence remains the conditional ablation of Wnt secretion in intestinal Foxl1-/Gli1-expressing subepithelial telocytes, which demonstrates their necessity for stem-cell niche maintenance. By contrast, most evidence from cardiac, dermal, reproductive, and fibrotic tissues remains supplementation-based or correlative. We propose a unified Stromal Network Organiser framework, in which telocytes are interpreted as tissue-specific stromal network cells whose dysfunction may involve not only numerical loss, but also telopode fragmentation, contact uncoupling, paracrine alteration, stromal replacement, and failed niche signalling. To improve methodological clarity, we introduce a two-dimensional evaluation matrix that separates identification confidence from functional confidence. We argue that the next decade of telocyte research should prioritise loss-of-function experiments outside the intestine, independent replication of foundational single-laboratory concepts, and high-resolution multi-organ correlative ultrastructural atlases.

## 1. Introduction

Telocytes are stromal cells characterised by a small cell body (typically 6–12 µm in its largest dimension) and extremely long, thin, moniliform prolongations termed telopodes, which range from tens to hundreds of micrometres in length and frequently exceed 100 µm, in which slender segments—podomeres, approximately 80 nm in width and frequently below the resolution of conventional light microscopy—alternate with dilated regions, termed podoms, containing mitochondria, endoplasmic reticulum, and caveolae. Telocytes should be understood as stromal cells with a mesenchymal-like phenotype, not as haematologic cells. Their partial overlap with fibroblasts and other stromal populations is a central reason why marker-based identification alone is insufficient, and we will discuss this in the following chapters. Their distinction from fibroblasts rests primarily on ultrastructural morphology, spatial organisation, long-range telopode connectivity, and comparatively limited matrix-producing features, rather than on a single exclusive immunophenotypic marker. They were progressively identified between 2005 and 2009 by the group of Popescu at the Carol Davila University of Medicine and Pharmacy, Bucharest, initially under the term interstitial Cajal-like cells (ICLC) [1,2,3,4], and were renamed telocytes in a 2010 consensus paper by Popescu and Faussone-Pellegrini [5]. Two decades later, telocytes have been described in essentially every mammalian organ [6,7,8]; morphologically comparable cells have since been reported across non-mammalian vertebrates, including birds, reptiles, amphibians, and fish [9,10,11,12], indicating that the telocyte phenotype is evolutionarily widespread rather than mammal-specific. Telocytes have been implicated, with varying degrees of evidence, in stem-cell niche regulation, intercellular communication via extracellular vesicles, and stromal alterations in fibrosis, inflammation, and cancer [13,14,15,16].

The maturation of the field has been uneven. Two principal advances have been consolidated. First, in the gastrointestinal tract, conditional ablation of Wnt secretion in subepithelial Foxl1-expressing telocytes—and in the partially overlapping Gli1-expressing mesenchymal population—was shown in 2018 to collapse intestinal stem-cell maintenance, providing the first in vivo loss-of-function evidence that a telocyte subpopulation is functionally indispensable for tissue homeostasis [17,18,19,20]. Second, a converging body of work, principally from the Florence school, has documented progressive telocyte depletion in systemic sclerosis [21,22] and analogous reductions across multiple fibrotic and inflammatory conditions [16,23,24,25,26,27]. Together, these two strands strongly support the conclusion that telocytes represent a distinct stromal entity whose alteration is associated with disease.

Several other claims, by contrast, remain at the level of plausible hypothesis. Cardiac telocyte transplantation experiments report functional benefit after experimental myocardial infarction [23,24], and conditioned-medium studies report effects on cardiac stem cells [28]; the skin telocyte secretome inhibits fibroblast-to-myofibroblast transition [29]; and endometrial telocyte-derived exosomes carry Wnt ligands that support regeneration [30]. These results, however, demonstrate that exogenously supplied telocyte products are biologically active rather than that endogenous telocytes are necessary for tissue function. The distinction between sufficiency and necessity is central to the field and has not yet been resolved outside the gut. A further group of foundational concepts—the stromal synapse [31], miR-193 as a discriminating molecular signature [32], and the three-class taxonomy of telocyte-derived extracellular vesicles [15,33]—has substantial conceptual appeal but has not, to our knowledge, been independently replicated in laboratories outside the originating group with the resolution that contemporary techniques would allow. These concepts are therefore best regarded as historically important hypotheses that merit dedicated reinvestigation rather than as established facts.

A persistent methodological problem is that, in much of the literature, telocytes are identified solely by CD34 immunoreactivity in spindle-shaped stromal cells, without ultrastructural confirmation, spatial network analysis, or functional perturbation [34,35]. CD34 is shared with a heterogeneous population of CD34-positive stromal fibroblasts/fibrocytes [34]; PDGFRα labels a broader subepithelial mesenchymal compartment [36]; and vimentin and c-kit are non-specific. Marker-based identification, on its own, is therefore insufficient for diagnosing telocyte identity, although it remains essential at scale. Minimal criteria for high-confidence telocyte identification are proposed in Supplementary Table S1. The corrective is not to abandon immunohistochemistry, but to anchor marker-based identification in supportive ultrastructural evidence and, where claims of function are made, in perturbation-based experiments.

This review is written from within the historical and conceptual development of the telocyte field. Its purpose is therefore both critical and constructive: to acknowledge the originality of telocyte discovery, including the foundational contributions of the Bucharest school, while applying the evidential standards required for the next phase of the field to this literature. We do not aim to provide another organ-by-organ catalogue of telocyte distribution—a task already attempted in earlier reviews [7,8,13,37]. Instead, we ask three questions. First, which claims in telocyte biology are robustly demonstrated, which remain biologically plausible but insufficiently tested, and which require independent re-examination with contemporary methods? Second, can the proliferation of partially overlapping interpretive models be consolidated into a single, testable framework? Third, what counter-evidence exists, and how should it delimit or modify that framework? On this basis, we propose two main contributions: a unified Stromal Network Organiser framework that consolidates dispersed models of telocyte function into a testable interpretive structure, and a two-dimensional evaluation matrix that separates confidence in telocyte identification from confidence in telocyte function. By combining a historical perspective with methodological self-critique, we seek to move the field beyond the question of whether telocytes exist or matter, toward the more precise questions of where they are functionally indispensable, through which molecular signals they act, and under what pathological conditions their network failure becomes biologically or clinically relevant.

### Methods and AI-Assisted Figure Preparation

The literature discussed in this review was selected to address the main evidential levels in telocyte research: original ultrastructural descriptions, organ-mapping studies, three-dimensional reconstruction studies, marker-based identification studies, analyses of stromal mimics, extracellular-vesicle and secretome studies, disease-associated observations, and functional perturbation experiments. Particular attention was given to separating descriptive, correlative, sufficiency-based, and necessity-based evidence.

Schematic figures were created and revised with FigureLabs (https://www.figurelabs.ai/; accessed on 20 May, 24 June and 4 July 2026), based on author-defined scientific instructions. FigureLabs was used only to assist in the preparation of conceptual schematic illustrations. It was not used to generate experimental data, histological images, electron-microscopy images, ultrastructural reconstructions, quantitative results, literature interpretation, or scientific conclusions.

The authors provided the scientific concepts, figure structure, cellular elements, terminology, labels, and interpretive framework for each figure. All FigureLabs-assisted outputs were reviewed, edited where necessary, and scientifically validated by the authors for consistency with the telocyte literature, anatomical plausibility, terminology, figure legends, and the manuscript text. The final figures are schematic conceptual illustrations and should not be interpreted as primary experimental images or direct ultrastructural reconstructions. The authors take full responsibility for the final content of all figures.

## 2. The Discovery Era: From ICLC to Telocytes

The history of telocytes cannot be separated from the broader history of interstitial cells of Cajal and from the question of whether Cajal-like stromal cells exist outside the gastrointestinal tract. Between 2005 and 2009, c-kit-positive interstitial cells with distinctive ultrastructural prolongations were described in several human organs, including the fallopian tube [1], myometrium [2], pancreas [3], atrial myocardium [4], ventricular myocardium [38], resting mammary gland [39], gallbladder [40], placenta [41], and additional tissues. The term interstitial Cajal-like cells was initially used because c-kit immunoreactivity, interstitial location, and elongated morphology evoked gastrointestinal interstitial cells of Cajal. This terminology was provisional. As evidence accumulated in extra-digestive organs, it became increasingly clear that these cells were not ectopic gastrointestinal ICC but a related yet distinct stromal phenotype.

**Figure 1:**
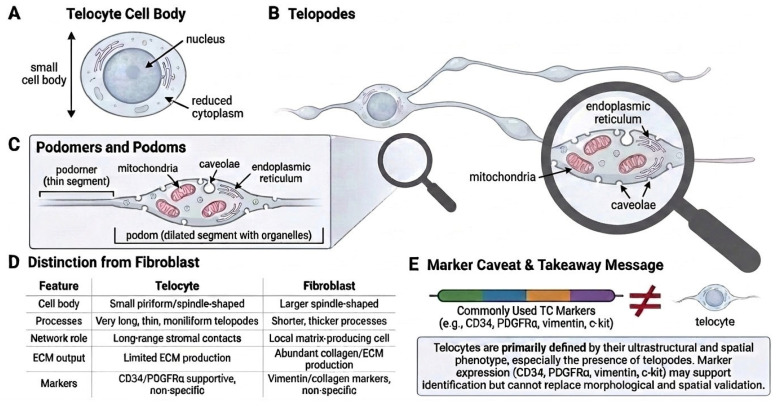
Defining the telocyte: morphology before markers. Telocytes are primarily defined by their ultrastructural and spatial phenotype rather than by a single immunohistochemical marker. Their defining features include a small cell body, extremely long and thin telopodes, and a moniliform architecture in which slender podomeres alternate with dilated podoms containing mitochondria, endoplasmic reticulum, and caveolae. Marker expressions such as CD34, PDGFRα, vimentin, or c-kit may support identification but cannot replace morphological and spatial validation. This distinction is essential for separating telocytes from fibroblasts, pericytes, endothelial-associated stromal cells, and other CD34-positive interstitial cells. The drawing is a schematic conceptual representation and should not be interpreted as a literal ultrastructural reconstruction; telocyte identity remains dependent on ultrastructural and spatial validation. Created and revised with FigureLabs (https://www.figurelabs.ai/), accessed on 20 May and 24 June 2026, and scientifically reviewed and validated by the authors.

In 2010, Popescu and Faussone-Pellegrini proposed the term telocyte to designate this cell population [5,42]. The corresponding term telopode was introduced to define the characteristic cellular prolongation: extremely long, extremely thin, and moniliform, with slender podomeres alternating with dilated podoms containing mitochondria, endoplasmic reticulum, and caveolae [43,44]. The subsequent conceptual consolidation of the terminology shifted the field from definition by analogy—“Cajal-like”—toward definition by cellular architecture. This transition was more than nominal: it provided a morphological identity for a stromal cell type that could be recognised across organs without requiring direct equivalence with gastrointestinal ICC. The defining morphological features of telocytes, and the reason why marker expression alone is insufficient for their identification, are summarised in Figure 1.

The conceptual transition from ICLC to telocytes and the subsequent evolution from organ mapping to network biology are summarised in Table 1.

Several methodological caveats are important when this discovery period is read retrospectively. First, the foundational ICLC papers relied primarily on c-kit immunoreactivity, light microscopy, and transmission electron microscopy, reflecting the methodological standards available at the time. According to the evaluation matrix proposed in this review, these studies would now be placed at low-to-moderate identification confidence, depending on the degree of ultrastructural support, and at low functional confidence, because they were principally descriptive rather than perturbation-based. Second, the original interpretation of these cells as Cajal-like implied possible pacemaker, neuromodulatory, or contractile-coordination roles in extra-digestive smooth-muscle organs [47], but most of these functions remained inferential rather than experimentally demonstrated. Third, the 2010 renaming clarified nomenclature and identity but did not by itself establish biological necessity.

**Figure 2:**
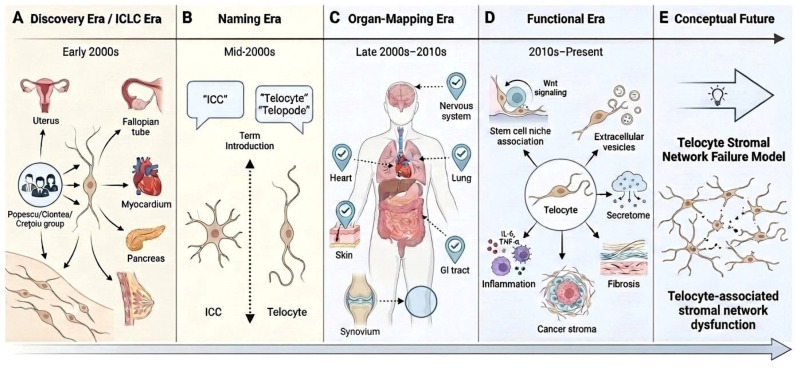
Evolution of telocyte research over two decades. The schematic provides a visual synthesis of the five conceptual phases detailed in Table 1, tracing the field from the early recognition of interstitial Cajal-like cells, through the formal telocyte/telopode terminology and widespread organ mapping, to recent functional studies. To complement rather than duplicate the tabular summary, the figure emphasises the overall trajectory rather than individual contributions. Its final panel illustrates the conceptual transition proposed in this review, from telocytes as organ-mapped stromal cells to telocyte-associated stromal network dysfunction. Created and revised with FigureLabs (https://www.figurelabs.ai/) accessed on 20 May, 24 June and 4 July 2026, and scientifically reviewed and validated by the authors.

These qualifications should not be interpreted as diminishing the originality of the discovery era. On the contrary, they make clear why the contribution was important: the early studies identified a recurrent stromal cell morphology that conventional histology had largely missed, introduced the problem of long-range stromal connectivity in multiple organs, and created the conceptual foundation for subsequent work on telocyte networks, paracrine signalling, and tissue microenvironment organisation. The discovery era should therefore be understood as the phase in which telocytes became morphologically visible and conceptually separable from ICC, fibroblasts, pericytes, and other interstitial cells; the functional meaning of that distinction became the task of the following two decades. The main historical and conceptual phases of telocyte research are summarised in Figure 2.

## 3. The Organ-Mapping Era and Its Limits

After the introduction of the telocyte/telopode terminology, reports of telocytes expanded rapidly across organs and species, including the heart [48,49], lung [50], gastrointestinal tract [35,51], urinary tract, skin [52], uterus and fallopian tube [53,54], placenta [41], skeletal muscle [55], synovium, pancreas [3], mammary gland [39], and peripheral nervous system [56]. The breadth of distribution supported the view that telocytes are a general component of tissue microenvironments rather than a tissue-specific curiosity.

Two principal weaknesses limited the cumulative scientific yield of this era. First, identification standards became heterogeneous as the literature expanded. In a substantial proportion of organ-mapping studies, telocytes were identified primarily through CD34, PDGFRα, PDGFRβ, vimentin, or c-kit immunoreactivity in spindle-shaped stromal cells, without TEM confirmation and without exclusion of mimics such as CD34-positive fibroblasts/fibrocytes, pericytes, and endothelial-associated stromal cells [34,57]. Because podomeres are extremely thin, approximately 80 nm in width, and frequently below the resolution of conventional light microscopy, immunofluorescence-based identification of “long CD34-positive processes” does not reliably establish telopode identity [58]. Second, functional inference often outpaced experimental demonstration. Anatomical proximity of telocytes to smooth muscle cells, vessels, nerves, immune cells, or progenitors was repeatedly interpreted as evidence of regulatory function, although such proximity is also expected for any widely distributed stromal cell.

The main stromal and interstitial mimics that must be distinguished from telocytes are compared in Table 2.

Notwithstanding these limitations, the organ-mapping era contributed three durable findings. It established that telocytes, or telocyte-like populations, are widely distributed; it generated tissue-specific morphological descriptions, particularly the three-dimensional networks documented in cardiac [45] and myometrial [46] tissues by FIB-SEM tomography and serial reconstruction; and it produced hypotheses that subsequent functional studies have begun to test. The principal lesson is that the question “are telocytes present in this organ?” was, by approximately 2015, no longer informative on its own. Future organ-specific work is most useful when it adds mechanistic or disease-relevant insight, or when it provides high-resolution three-dimensional reconstructions beyond the reach of routine microscopy. In particular, contrary to claims sometimes made in earlier reviews [8], the field is not yet replete with comprehensive multi-organ ultrastructural atlases obtained with modern correlative microscopy techniques. Such atlases remain a legitimate goal for the next decade. The principal methodological pitfalls that have contributed to overinterpretation in the field are summarised in Supplementary Table S2.

### Practical Hardware and Experimental Requirements for Telocyte Identification

For readers outside the telocyte field, a practical distinction is essential: telocytes are not defined by a single marker, but by the convergence of morphology, spatial organisation, marker support, and, where function is claimed, experimental perturbation. Telocytes are stromal cells with a mesenchymal-like phenotype; they are not haematologic cells. They share several markers with fibroblasts and other stromal populations, including CD34, PDGFRα/β, vimentin, and caveolin-1, but they differ from conventional fibroblasts in their very small cell body, extremely long and thin telopodes, moniliform architecture, long-range stromal connectivity, and limited matrix-producing phenotype.

The “hardware” required for high-confidence telocyte identification therefore includes transmission electron microscopy to visualise telopodes, podomeres, podoms, caveolae, mitochondria, and endoplasmic reticulum; serial ultrathin sectioning, FIB-SEM, or serial block-face SEM to reconstruct three-dimensional morphology; immunohistochemistry or immunofluorescence to provide marker support; and spatial or correlative microscopy to distinguish telocytes from fibroblasts, pericytes, endothelial-associated stromal cells, interstitial cells of Cajal, and immune cells. Marker expression alone should be considered supportive but not diagnostic.

A corresponding experimental “to-do list” follows from these limitations. Future studies should: (i) combine marker-based identification with ultrastructural or three-dimensional validation; (ii) explicitly compare telocytes with fibroblasts and other stromal mimics in the same tissue; (iii) distinguish observed telocyte loss or network disruption from theoretical dysfunction; (iv) identify the underlying disease context in which telocyte alterations are reported; and (v) use causal perturbation approaches, such as lineage-specific ablation, conditional signalling deletion, rescue experiments, or spatially resolved functional assays, before assigning indispensable biological roles to telocytes outside the intestinal Wnt niche. These practical methodological requirements are summarised in Table 3.

## 4. The Functional Evidence: What Is Demonstrated and What Is Proposed

Functional studies of telocytes vary substantially in evidential strength, and the field is not well-served by a uniform reading of this literature. We organise the evidence by tissue, distinguishing where possible between experiments that demonstrate necessity (loss-of-function in vivo, with phenotypic rescue) and those that demonstrate *sufficiency* (addition of telocyte-derived material, or co-culture, with a measured effect).

### 4.1. Intestine: The Strongest Available Evidence

The most rigorous functional evidence emerges from the gastrointestinal tract. In two independent 2018 *Nature* studies, conditional deletion of *Porcn* (which abolishes Wnt secretion) in subepithelial *Foxl1*-expressing telocytes—and in the partially overlapping *Gli1*-expressing mesenchymal population—produced rapid collapse of crypt proliferation and loss of intestinal stem-cell function [17,18]. These experiments demonstrate *necessity*: in their absence as a Wnt source, the intestinal stem-cell compartment fails. The findings have been consolidated in subsequent commentary and extended in further work [19,20]. They also raise an important interpretive caveat. The studies identified the relevant cells through molecular markers (Foxl1, Gli1) and lineage tracing, not through systematic TEM verification of telopodes. Whether *all* Foxl1- or Gli1-positive subepithelial mesenchymal cells correspond exactly to the classical ultrastructural telocyte or whether they constitute a partially overlapping but molecularly distinct subset has not been definitively resolved. This is not a methodological criticism of the original studies; it is, however, a reason to be cautious before extrapolating the intestinal paradigm to other tissues without comparable evidence.

### 4.2. Heart, Skin, Endometrium: Sufficiency Without Necessity

Outside the intestine, the available evidence is largely supplementation-based. In the heart, intramyocardial transplantation of cardiac telocytes after experimental infarction reduced the infarct size and improved post-infarct function [23,24]; conditioned medium from cardiac telocytes modulated cardiac stem-cell behaviour [28,62], consistent with the early ultrastructural localisation of telocytes within epicardial stem-cell niches alongside cardiomyocyte precursors [63]. In the skin, conditioned medium from cultured skin telocytes prevented profibrotic differentiation of dermal fibroblasts into myofibroblasts [29]. In the endometrium, exogenously supplied telocyte-derived exosomes carrying Wnt ligands inhibited fibrosis and supported regeneration in injury models [30]. Each of these results is biologically informative; cumulatively, they establish that telocytes—or material derived from them—possess regulatory activity. None demonstrate that endogenous telocytes are *required* for tissue function in the corresponding organ. The intestinal *Porcn* deletion paradigm has not yet been replicated, to our knowledge, in heart, skin, or reproductive tissues. Until comparable conditional ablation experiments are performed, claims that telocytes “maintain” cardiac, dermal, or reproductive homeostasis must be qualified accordingly.

### 4.3. Conclusions from the Functional Evidence

The functional literature thus permits one robust conclusion (telocytes are necessary for intestinal stem-cell maintenance via Wnt secretion) and a series of plausible but unconfirmed extensions (telocyte-derived signals influence repair, fibrosis, and immune balance in other tissues). The robust conclusion supports the broader interpretive framework that telocytes act as paracrine niche cells. The plausible extensions motivate further functional work, particularly conditional ablation experiments, but should not, on present evidence, be promoted to the same evidential status as the intestinal finding. A tissue-by-tissue critical interpretation of the current telocyte evidence is provided in Table 4.

## 5. Telocytes as Stromal Network Cells: A Calibrated Synthesis

**Figure 3:**
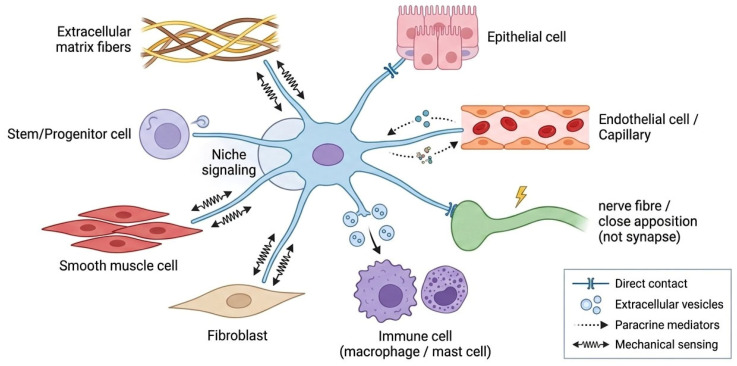
Telocytes as stromal network cells. Telocytes may function as stromal network cells by connecting epithelial, vascular, neural, immune, fibroblastic, smooth-muscle, and progenitor compartments through telopodes, direct contacts, extracellular vesicles, paracrine mediators, and possibly mechanical sensing. This schematic illustrates the architectural basis for the Stromal Network Organiser framework, while acknowledging that many functional consequences of these interactions remain to be demonstrated experimentally. Created and revised with FigureLabs (https://www.figurelabs.ai/), accessed on 20 May and 24 June 2026, and scientifically reviewed and validated by the authors.

A useful description of telocytes that integrates morphological, distributional, and functional evidence is that they are stromal cells whose telopodes extend over long distances and establish multiple contacts within the tissue microenvironment. Three-dimensional reconstructions obtained by FIB-SEM tomography of human cardiac [45] and skin telocytes [44] and serial ultrastructural reconstruction of the human myometrial telocyte network [46] confirm that telocytes form continuous interstitial networks that are underrepresented by two-dimensional histology. The architecture is consistent with a role in providing structural continuity within the interstitium and in interfacing with multiple neighbouring cell types. This network cell interpretation is illustrated schematically in Figure 3.

Beyond this descriptive consensus, several inferences about the functional consequences of network organisation should be treated as working hypotheses rather than facts. The proposal that telocytes “create microdomains of communication”, “organise tissue compartments”, or “coordinate the spatial distribution of regenerative or inflammatory signals” [15,68] is plausible and consistent with the known morphology, but a direct experimental demonstration that disruption of network topology—without altering cell number—alters tissue physiology is not yet available for any organ. A useful definition of telocytes, which we adopt in this review, is therefore that stromal cells characterised by telopodes (with podomeres and podoms), extensive long-range contacts with other cell types, and tissue-specific molecular profiles. This definition is operational and avoids the two opposite errors of reducing telocytes to a single marker and of treating them as identical across organs. It does not, in itself, ascribe function.

A direct consequence of this definition is that telocyte identification cannot rely on dissociated-cell methods alone. Single-cell RNA sequencing identifies stromal molecular clusters but destroys telopode architecture. The identification of telocytes requires methods that preserve or reconstruct spatial context—TEM, serial block-face SEM, FIB-SEM tomography, super-resolution and confocal microscopy, multiplex immunostaining, and spatial transcriptomics. Single-cell sequencing is complementary to, not a substitute for, these approaches [61]. As a corollary, claims that a transcriptomic stromal cluster “is” a telocyte should be regarded as provisional pending spatial and ultrastructural validation.

## 6. Intercellular Communication: Demonstrated Mechanisms and Single-Laboratory Hypotheses

Telocytes contact and may influence neighbouring cells through several modes that vary substantially in evidential strength. Direct intercellular contacts of multiple morphological types have been documented at the ultrastructural level, including gap junctions, puncta adhaerentia minima, processus adhaerens, and atypical close appositions whose biophysical properties remain incompletely characterised [59,60]. Cardiac telocyte junctional descriptions by Gherghiceanu and Popescu [59] provide the most systematic account; other organ-specific descriptions broadly support the picture of structural connectivity through a heterogeneous junctional repertoire.

Among the more distinctive concepts of the founding period is the “stromal synapse” [31]—a structurally specialised, signalling-competent contact between a telocyte and a non-telocyte partner, originally proposed based on TEM observations of narrow intercellular cleft, focal pre- and post-synaptic densities, and frequent vesicle clustering on the telocyte side. This concept has substantial conceptual appeal, but its current evidential status is limited. To our knowledge, the existence of polarised pre- and post-synaptic machinery—comparable to the molecular organisation of the neuronal synapse—has not been independently verified by super-resolution microscopy, expansion microscopy, or spatial proteomics in laboratories outside the originating group. The structures described in [31] could correspond either to specialised signalling synapses or to non-polarised close appositions without dedicated molecular machinery. The stromal synapse is therefore best regarded as a historically important hypothesis whose experimental verification with contemporary high-resolution methods remains an open priority. We make this point not to dismiss the concept but to align its citation status with the available evidence.

Telocyte-derived extracellular vesicles have been characterised in early Bucharest studies [15,33], which described three classes—exosomes (~45 nm), ectosomes (~130 nm), and shed vesicles—and in subsequent work on human skin telocytes by FIB-SEM tomography [44]. The three-class classification preceded the broader extracellular vesicle taxonomy and has not, to our knowledge, been independently re-examined with contemporary nano-flow cytometry, single-vesicle imaging, or proteomic profiling specifically on telocyte-derived vesicles in laboratories outside the originating group. Functional studies of telocyte-derived vesicles, when they have been performed, have demonstrated biological activity: cardiac telocyte secretomes modulate cardiac stem-cell behaviour [28]; endometrial telocyte-derived exosomes deliver Wnt ligands that support regeneration [30]; and skin telocyte secretomes prevent fibroblast-to-myofibroblast transition [29]. These results establish the biological activity of telocyte-derived material; they do not, on their own, validate the original three-class vesicle taxonomy.

A further concept that requires recalibration is the proposal that miR-193 constitutes a discriminating molecular signature of telocytes [32]. The original report compared telocytes against fibroblasts in cardiac tissue and identified differential miR-193 expression. The result is interesting and compatible with a tissue-specific molecular signature, but to our knowledge it has not been independently replicated in multiple tissues by laboratories outside the originating group, and it has not been benchmarked against the much larger set of stromal markers that contemporary single-cell transcriptomics now makes available. The claim that miR-193 represents a “discriminating signature” should therefore be treated as a single-laboratory observation in need of replication; it should not be cited as if it had the standing of a validated cell-type marker.

Taken together, the evidence on telocyte communication supports a calibrated synthesis. Telocytes participate in a heterogeneous intercellular contact repertoire and release extracellular vesicles with measurable biological activity. The detailed molecular organisation of these contacts (whether they include polarised stromal synapses) and the molecular taxonomy of the vesicles (whether the original three-class scheme remains the most informative description) require independent re-examination with contemporary techniques. Candidate mediators reported across tissue contexts—secreted ligands, vesicle cargo, and contact-dependent signals—are discussed below as mechanistic hypotheses within the SNO framework. Functional consequences of telocyte communication—including the proposal that telocytes provide a “paracrine field” that restrains excessive fibroblast activation [29]—are presented in this review (Section 8) as components of an interpretive framework rather than as established mechanisms.

A recent series of studies has extended these communication hypotheses to intestinal and fascial inflammation. Working in experimental colitis models, a single group has proposed that CD34+/PDGFRα+ telocytes participate in a telocyte–macrophage “force–immune axis”, responding to mechanical and acupuncture stimuli and promoting M2 macrophage polarisation together with anti-inflammatory Wnt/Fgf signalling [69,70,71]. These reports are mechanistically suggestive and methodologically ambitious, combining transmission electron microscopy with single-cell RNA sequencing and deep-learning analysis. They nonetheless share two limitations that recur throughout this review: all three originate from the same laboratory, and their central mechanistic claims rest substantially on computational inference rather than on direct loss-of-function perturbations. They are therefore best regarded, at present, as promising single-laboratory hypotheses that require independent replication and in vivo causal testing before the proposed force–immune axis can be considered established.

## 7. The Female Reproductive Tract: Hormonally Responsive Stroma and Open Functional Questions

The female reproductive tract has historical importance for the field—the early studies on the human fallopian tube [1] and myometrium [2] contributed substantially to the recognition of telocytes as a distinct stromal population—and it continues to be of biological interest because the relevant tissues combine endocrine responsiveness, smooth muscle activity, cyclic regeneration, and clinically important fibrotic and inflammatory disorders. The available evidence supports several specific points.

First, telocytes in human fallopian tubes express oestrogen and progesterone receptors [65], providing direct evidence of hormonal responsiveness. Three-dimensional ultrastructural mapping of human myometrial telocyte networks [46] shows that these cells form continuous interstitial networks connecting longitudinal and circular muscular layers, vessels, and nerves. There is a pharmacological observation that imatinib—a tyrosine-kinase inhibitor that blocks c-kit (CD117), PDGFRα/β, and BCR-ABL signalling—inhibits spontaneous rhythmic contractions of the human uterus and intestine [64] provides circumstantial functional evidence linking c-kit-expressing interstitial cells to contractile activity, although it does not specifically isolate the telocyte contribution from that of other c-kit-positive populations.

**Figure 4:**
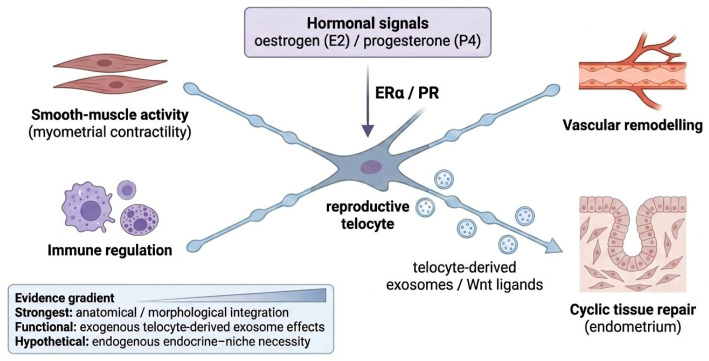
The reproductive telocyte endocrine-niche model. In the female reproductive tract, telocytes may act as endocrine-responsive stromal network cells integrating hormonal signals, smooth-muscle activity, vascular remodelling, immune regulation, and cyclic tissue repair. Evidence is strongest for anatomical and morphological integration in the fallopian tube and myometrium and for exogenous telocyte-derived vesicle effects in endometrial repair models. Broader claims of endogenous endocrine-niche necessity remain hypotheses requiring direct loss-of-function testing. This schematic represents a conceptual endocrine–stromal integration model and not a direct experimental reconstruction. Created and revised with FigureLabs (https://www.figurelabs.ai/), accessed on 20 May and 24 June 2026, and scientifically reviewed and validated by the authors.

Second, in the endometrium, telocyte-derived exosomes carrying Wnt ligands have been shown to inhibit fibrosis and support regeneration in injury models [30]. As with the analogous cardiac and skin work (Section 4.2), this is a sufficiency demonstration: exogenously supplied telocyte-derived material has biological effects. Whether endogenous endometrial telocytes are required for cyclic regeneration has not been tested by conditional ablation. Several questions therefore remain open and would benefit from direct experimental investigation: whether telocyte morphology or density varies systematically across the menstrual cycle and pregnancy; whether telocyte-related parameters are altered in endometriosis, adenomyosis, intrauterine adhesions, or unexplained infertility; whether telocyte–macrophage and telocyte–mast cell interactions in reproductive tissues parallel those described in the gut [72,73]; and whether endogenous telocyte secretion is necessary for scarless endometrial repair. These anatomical, endocrine, and regenerative hypotheses are integrated schematically in Figure 4.

From the present evidence, it is reasonable to describe reproductive-tract telocytes as hormonally responsive stromal network cells with documented anatomical and morphological features and a single rigorous functional demonstration via exogenous exosome supplementation. Broader claims of integrated endocrine-niche function are interpretively attractive but await direct experimental support.

## 8. A Unified Stromal Network Organiser Framework

The recent literature has proposed several partially overlapping interpretive models for telocyte function in homeostasis and disease, including network failure, paracrine shielding, telocyte–myofibroblast balance, immune rheostat function, reproductive endocrine integration, and stromal boundary maintenance [15,74,75]. These models and their testable predictions are summarised in Supplementary Table S4. Examined critically, they share a common conceptual core: telocytes are proposed to organise stromal microenvironments through long-range physical contacts, paracrine signalling, extracellular vesicle release, and heterocellular interactions, while disease is proposed to result from disruption of this organisation. Their differentiation by name is, in our view, less useful than their integration into a single, testable framework. We therefore consolidate these overlapping proposals into the Stromal Network Organiser (SNO) framework, expressed through tissue-specific sub-themes and explicitly bounded by the available evidence.

### 8.1. The Unified Framework

The framework rests on three connected propositions, each labelled with the highest grade of evidence currently available.

**Proposition** **1** **(descriptive; well supported).** *Telocytes form long-range stromal networks via telopodes, with documented heterocellular contacts to multiple cell types of different lineages. This proposition is supported by TEM and FIB-SEM evidence in cardiac [45], skin [44], and myometrial [46] tissues, as well as consistently in multiple other organs [43,59,60]. It is among the more robustly established statements in the field.*

**Proposition** **2** **(mechanistic; partially supported).** *Telocyte-derived signals—including secreted ligands such as Wnt and material delivered by extracellular vesicles—can influence neighbouring stem cells, fibroblasts, endothelial cells, and immune cells. This proposition is robustly supported in the intestine [17,18]; moderately supported by sufficiency-based studies in the heart [23,24,28], skin [29], and endometrium [30]; and weakly supported elsewhere. The proposition that telocytes are necessary for these functions outside the intestine is currently unverified.*

**Proposition** **3** **(pathological; interpretive).** *Disorders of stromal organisation—fibrosis, chronic inflammation, repair failure, and cancer-associated stromal disorganisation—may, in some tissues and disease contexts, involve disruption of telocyte networks. This proposition is descriptively supported by reproducible reports of decreased telocyte density in fibrotic and chronic inflammatory tissues [16,21,22,27], and mechanistically supported in vitro by the antifibrotic effect of skin telocyte secretome [29]. Its causal interpretation in vivo is not yet established and is subject to important counter evidence (Section 8.4).*

### 8.2. Tissue-Specific Sub-Themes

Wnt-niche support (intestine). Subepithelial Foxl1-expressing telocytes are a necessary Wnt source for intestinal stem-cell maintenance [17,18]. This is the strongest available demonstration of telocyte function, and the principal anchor of the framework.

Antifibrotic regulation (skin, lung, others). Telocyte secretome inhibits profibrotic differentiation of dermal fibroblasts in vitro [29]. Telocyte depletion in fibrotic skin and other organs is reproducibly observed in human disease [21,22]. Whether endogenous telocyte signalling restrains fibrosis in vivo awaits conditional ablation evidence.

Immune calibration (gut, others). Co-culture studies and integrative reviews suggest that telocyte–macrophage interactions modulate macrophage morphology, junctional behaviour, and apoptosis [72,73]. These results support an immune-modulatory role; their direction (anti-inflammatory, pro-resolving, or context-dependent) is not uniform and likely depends on the tissue and stage.

Reproductive endocrine integration. Hormone-receptor expression [65] and architectural integration with smooth muscle and vasculature [46] are documented; functional necessity for cyclic remodelling is not.

### 8.3. Telocyte-Associated Stromal Network Dysfunction as Network-Level Dysfunction

**Figure 5:**
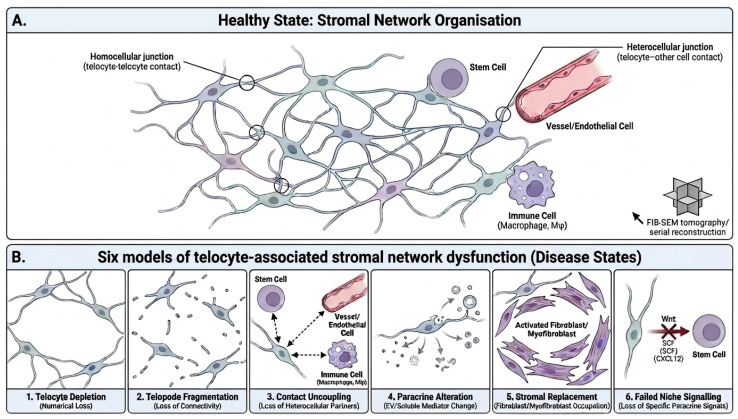
The Stromal Network Organiser framework and six modes of telocyte-associated stromal network dysfunction. (**A**) In the healthy state, telocytes form long-range stromal networks via telopodes, with documented homocellular junctions between adjacent telocytes and heterocellular contacts with stem cells, vessels, and immune cells (Mφ, macrophage). This architecture has been visualised by FIB-SEM tomography in cardiac and skin tissue and by serial reconstruction in human myometrium [44,45,46]. (**B**) Six non-exclusive modes by which the network may fail in disease: (1) telocyte depletion, or numerical loss; (2) telopode fragmentation, with loss of long-range connectivity despite preserved cell bodies; (3) contact uncoupling, with loss of specific heterocellular partners; (4) paracrine alteration, involving changes in extracellular vesicle release, vesicle cargo, or soluble mediator output; (5) stromal replacement, with occupation of telocyte territory by activated fibroblasts or myofibroblasts; and (6) failed niche signalling, including loss of Wnt or other paracrine signals to resident stem-cell compartments. Counter-evidence and boundary conditions of the framework are discussed in Section 8.4. Created and revised with FigureLabs (https://www.figurelabs.ai/), accessed on 20 May and 24 June 2026, and scientifically reviewed and validated by the authors.

Within the SNO framework, telocyte-associated disease is best conceived as network-level dysfunction rather than as the mere numerical loss of a cell type. Six non-exclusive modes of disruption can be specified, each amenable to experimental testing: telocyte depletion, defined as reduction in cell number through apoptosis, senescence, oxidative injury, or stromal replacement; telopode fragmentation, defined as loss of long-range connectivity even when cell bodies are preserved; contact uncoupling, defined as loss of specific heterocellular contacts with stem cells, vessels, immune cells, or smooth muscle cells; paracrine alteration, defined as changes in extracellular vesicle release, vesicle cargo, or soluble mediator output; stromal replacement, defined as occupation of telocyte territory by activated fibroblasts or myofibroblasts; and failed niche signalling, defined as loss of specific paracrine signals, such as Wnt, to resident stem-cell compartments. These categories are not mutually exclusive; in human disease, they are likely to overlap. The proposed network-level interpretation of telocyte-associated stromal network dysfunction within the Stromal Network Organiser framework is summarised in Figure 5.

Mechanistic specificity is essential if the SNO framework is to remain testable rather than metaphorical. In the intestine, loss of telocyte-derived Wnt signals, including Wnt2b and Rspo3 from Foxl1-expressing stromal cells, leads to failure of Lgr5-positive intestinal stem-cell maintenance [17,18]. By analogy, loss of telocyte-derived antifibrotic or TGF-β-modulating signals in the skin would be expected to permit fibroblast-to-myofibroblast transition, a hypothesis supported at the level of sufficiency by skin telocyte secretome data [29]. Similarly, alteration of telocyte-derived extracellular vesicles or immune-modulatory signals could plausibly affect macrophage behaviour [72,73]. These molecule-level interpretations are presented as falsifiable hypotheses, not as established mechanisms outside the intestine.

### 8.4. Counter-Evidence and Boundary Conditions

A balanced framework requires explicit consideration of counter evidence. Several observations limit the generality of the SNO framework as currently formulated. Disease contexts in which telocyte alterations have been proposed, together with the corresponding evidential cautions, are summarised in Supplementary Table S5.

First, the relationship between telocyte density and fibrosis is not uniformly inverse. Although reproducible reports document telocyte loss in dermal fibrosis of systemic sclerosis [21,22] and analogous reductions in other fibrotic or chronic inflammatory conditions [16,27,76], evidence in some tissues, including certain cardiac settings [77], is more variable. In many disease contexts, the temporal relationship between telocyte alteration and matrix accumulation remains unresolved [78]. Telocyte loss may therefore be a cause, a consequence, or an epiphenomenon of tissue remodelling, depending on the organ and disease stage.

Second, numerical telocyte loss is not sufficient to define telocyte-associated stromal network dysfunction. A tissue may show telopode fragmentation, loss of heterocellular contacts, altered paracrine signalling, or immune dysregulation despite preserved telocyte counts. Conversely, reduced telocyte density does not automatically prove causal involvement in fibrosis or chronic inflammation. The available human studies are predominantly cross-sectional, and the relative scarcity of negative or non-confirmatory reports may reflect publication bias. For this reason, the preservation of telocyte density in established disease, fibrosis without prior telocyte alteration, or telocyte loss without fibrosis should be actively reported rather than treated as uninformative.

Third, the molecular identity of stromal cells implicated in disease remains heterogeneous. The CD34-positive stromal compartment includes telocytes, fibroblast-like cells, fibrocytes, perivascular stromal cells, and other interstitial populations whose lineage relationships are incompletely resolved [34]. Without lineage tracing, fate mapping, or spatial transcriptomic integration with ultrastructure, the attribution of disease-associated stromal changes specifically to telocytes remains presumptive. This caveat is particularly important in tumour stroma, where telocyte-like cells must be distinguished from cancer-associated fibroblasts and broader CD34-positive stromal remodelling [66,67].

These limitations do not refute the SNO framework, but they define its current evidential boundaries. The framework is strongest in the intestine, where conditional loss of Wnt secretion demonstrates functional necessity [17,18]; this is plausible but incompletely proven in skin and fibrotic disorders, where secretome and disease-association studies support a regulatory role [21,22,29], and provisional in cancer and other settings where the evidence remains predominantly correlative. We therefore present the SNO framework not as a settled theory, but as a disciplined interpretive structure designed to generate testable predictions.

Throughout this review, we distinguish between observed telocyte-associated alterations and theoretical dysfunctions inferred from network biology. Observed alterations include reduced telocyte density, telopode loss or fragmentation, and stromal replacement reported in defined disease contexts such as systemic sclerosis and other fibrotic or inflammatory tissues. By contrast, broader concepts such as impaired paracrine field organisation, failed immune calibration, disrupted endocrine-niche integration, or altered stromal boundary maintenance remain interpretive models unless supported by direct experimental perturbation in the relevant tissue.

## 9. A Two-Dimensional Evaluation Matrix for Telocyte Studies

Earlier proposals for evidence grading in telocyte research, including our own initial draft of this manuscript, have used a single linear scale that conflates identification confidence with functional confidence. In practice, these are independent dimensions: a study may identify a telocyte population through robust marker-and-lineage evidence without performing systematic TEM, while another may achieve excellent ultrastructural identification without any functional perturbation. A linear scale that requires TEM verification of telopodes before any functional study can be regarded as “strong” would, paradoxically, fail to give appropriate weight to the *Nature* loss-of-function studies in the gut [17,18]. We therefore propose a two-dimensional matrix, in which identification confidence and functional confidence are assessed separately. The structure of this matrix is shown in Table 5.

### 9.1. Identification Confidence (Rows of the Matrix)

I-3 (high). Telopode ultrastructure documented by TEM (showing podomeres and podoms), with spatial integration into stromal networks demonstrated; mimics excluded by appropriate marker panel.

I-2 (moderate). Marker-and-lineage identification (e.g., Foxl1 reporter, Gli1 lineage tracing, multiplex CD34/PDGFRα with morphological consistency), with at least suggestive ultrastructural support, with mimics considered.

I-1 (low). Single-marker identification (e.g., CD34 alone) in spindle-shaped stromal cells, without ultrastructural confirmation and without exclusion of mimics.

### 9.2. Functional Confidence (Columns of the Matrix)

F-3 (high). In vivo loss-of-function (conditional ablation, lineage-traced perturbation) with phenotypic rescue, demonstrating necessity.

F-2 (moderate). Sufficiency demonstration: addition of telocyte-derived material (cell transplantation, conditioned medium, isolated extracellular vesicles) producing a measurable phenotypic effect in vivo or in physiologically relevant models, or robust co-culture with rescue/perturbation.

F-1 (low). Descriptive association: spatial proximity, density change in disease tissue, and marker-shift correlation, without perturbation-based testing.

F-0 (none). Anatomical or descriptive identification only, with no functional component.

### 9.3. Application to the Literature

Applied to the principal studies of the field, the matrix produces a calibrated reading. The intestinal Porcn deletion experiments [17,18] are I-2/F-3: identification by Foxl1/Gli1 reporters with morphological consistency and partial ultrastructural support, with function demonstrated by conditional loss of necessity. They are the strongest available studies. The cardiac telocyte transplantation experiments [23,24] are I-2/F-2: marker-and-morphology identification of donor cells, with function shown by sufficiency in an injury model. The skin secretome studies [29] are I-2/F-2 with the additional caveat that the secretome derives from cultured cells whose telocyte identity is established by source-tissue dissection and morphology rather than by lineage tracing. The endometrial exosome work [30] is I-2/F-2.

Applied retrospectively to the foundational ICLC studies between 2005 and 2009, the matrix gives I-1 to I-2/F-1: marker-based identification with suggestive ultrastructural and pharmacological support, and descriptive associations rather than perturbation-based functional evidence. This is not a criticism of those studies; their methodological standards reflected the state of the field at the time, and they were essential for establishing telocytes as a recognisable cell population. It is, however, an honest acknowledgement that the founding studies do not, by contemporary criteria, themselves establish necessity. The same applies to the original “stromal synapse” [31] and miR-193 [32] reports, which, by the present criteria, are I-1/F-1 single-laboratory observations and would benefit substantially from independent replication.

We apply this matrix consistently throughout this review. The aim is to align evidential expressions with the strength of the available data, not to disqualify older or less perturbation-rich studies. Many essential findings in stromal biology are descriptive; what matters is that descriptive findings are presented as such, and that mechanistic claims are presented with their evidential basis stated.

### 9.4. Prospective Evidential Standards for Specific Claims

Beyond its retrospective application, the matrix can serve as a prospective standard for what evidence is required to support specific kinds of claims. Examples of strong versus weak formulations of telocyte claims are provided in Supplementary Table S3.

We propose the following minimal thresholds, intended for adoption by the broader telocyte research community:

(i) Telocyte identity in a new tissue. A claim that telocytes are present in a previously uncharacterised tissue should be considered provisional at I-1 (single-marker identification, e.g., CD34 alone) and convincing only at I-2 or above (marker-and-lineage with morphological consistency, ideally with at least suggestive ultrastructure). I-1-only claims should be reported with the explicit qualifier “telocyte-like” or “putative telocyte” until I-2 evidence becomes available.
(ii) Telocyte necessity for a function. A claim that telocytes are necessary for a particular tissue function requires F-3 evidence (in vivo loss-of-function with phenotypic rescue), irrespective of the identification confidence achieved. Sufficiency studies (F-2), however thorough, do not establish necessity, and language asserting that telocytes “maintain” or “are required for” a given homeostatic function should be reserved for tissues where F-3 evidence exists. At present, this threshold is met only for intestinal Wnt-niche support.
(iii) Telocyte sufficiency for a regenerative or modulatory effect. A claim of sufficiency—for example, that telocyte-derived exosomes can support endometrial regeneration, or that cardiac telocyte transplantation reduces infarct size—is acceptably supported at F-2 (in vivo or physiologically relevant supplementation with measured phenotypic effect), provided that I-2 identification of the source cells is documented. F-2 sufficiency claims should be reported as such and should not be conflated with F-3 necessity claims.
(iv) Telocyte involvement in disease pathogenesis. A claim that telocyte alteration contributes causally to a disease (rather than merely being associated with it) requires F-2 or F-3 evidence in a relevant disease model, in addition to F-1 association in human tissue. F-1 cross-sectional descriptive associations on their own should be reported as associations, not as causal implications.
(v) Independent replication of foundational concepts. Claims based on single-laboratory observations—currently including the stromal synapse, the three-class extracellular vesicle taxonomy, and miR-193 as a discriminating signature—should be cited with explicit acknowledgement of their replication status until contemporary independent re-examination is published.

We acknowledge that adoption of these standards by the field is voluntary, and that they may need refinement as new methods become available. Their value, in our view, is principally to provide explicit and shared expectations against which both authors and reviewers may calibrate the strength of claims.

**Figure 6:**
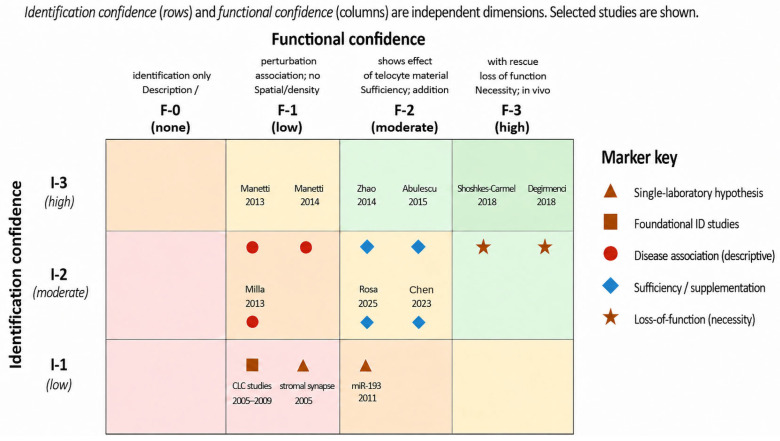
Two-dimensional evaluation matrix for telocyte studies. Rows indicate identification confidence (I-1, marker-based identification only; I-2, marker and lineage evidence with morphological consistency; I-3, ultrastructural validation with relevant cellular mimics excluded). Columns indicate functional confidence (F-0, description only; F-1, spatial or density association; F-2, sufficiency demonstrated through the addition, transplantation, secretome, or extracellular-vesicle material derived from telocytes; F-3, necessity demonstrated by in vivo loss-of-function experiments). The two dimensions are independent and are evaluated separately, thereby avoiding the conflation of cellular identification and functional evidence inherent in earlier linear evidence hierarchies. Selected landmark studies are positioned on the matrix to illustrate its application. The studies by Shoshkes-Carmel et al. (2018) [17] and Degirmenci et al. (2018) [18] occupy I-3/F-3, representing the highest combined identification and functional confidence currently illustrated in the field. Zhao et al. (2014) [24] and Abulescu et al. (2015) [28] occupy I-3/F-2, reflecting strong cellular identification together with evidence of functional sufficiency. Rosa et al. (2025) [29] and Chen et al. (2023) [30] occupy I-2/F-2. The disease-association study by Milla et al. (2013) [16] occupies I-2/F-1, whereas the studies by Manetti et al. (2013, 2014) [21,22] are positioned at I-3/F-1. The foundational ICLC studies published between 2005 and 2009 [1,2,3,4] and the original stromal synapse description from 2005 [31] occupy I-1/F-1. The miR-193 study from 2011 [32] occupies I-1/F-2, reflecting a potentially discriminating molecular signature that still requires broader independent validation. Marker placement represents the authors’ interpretation of the available literature; minor differences in classification are expected and may stimulate further discussion. Prospective evidential standards derived from this matrix are detailed in Section 9.4. Created with FigureLabs, accessed on 20 May 2026, and scientifically reviewed and validated by the authors.

The conceptual structure and representative placement of landmark telocyte studies within this matrix are shown in Figure 6.

## 10. Boundary Conditions: The Telocyte Controversy and Constructive Scepticism

Despite two decades of publication, the telocyte concept has not achieved universal acceptance in mainstream stromal or cell biology. This limited adoption reflects several unresolved issues: the absence of a unique molecular signature; frequent reliance on non-specific markers such as CD34, PDGFRα, vimentin, and c-kit; overlap with fibroblast-like stromal compartments; and incomplete integration of ultrastructural telocyte biology with single-cell and spatial transcriptomic frameworks [34,35,61]. The central unresolved question is therefore not whether telocyte-like cells exist, but whether telocytes represent a distinct stromal cell type, a specialised network-forming stromal phenotype, or a morphological state within broader CD34-positive fibroblast-like compartments.

This controversy should be addressed directly rather than avoided. Critics have reasonably argued that many reported telocytes, particularly when identified only by CD34 immunoreactivity, may correspond to heterogeneous CD34-positive stromal cells, fibrocytes, or fibroblast-like populations [34]. Conversely, the ultrastructural evidence for extremely long, moniliform prolongations, the reproducible observation of telocyte-like networks across organs, and the functional necessity of Foxl1/Gli1-associated Wnt-producing stromal cells in the intestine indicate that the field cannot be reduced to a simple artefact of marker interpretation [17,18,44,45,46]. The unresolved issue is whether morphology, spatial network behaviour, molecular identity, and functional necessity converge in the same cell population across tissues.

A related and very recent question concerns the relationship between telocytes and the CD34-positive “membranous cells” (CMCs) recently described in subcutaneous fascia by Huang and colleagues [79]. That study characterised broad, mechanosensitive CD34+ laminar membranous structures in vivo and in vitro and advanced a stereological argument that a two-dimensional line seen in a thin histological section can correspond to a three-dimensional laminar sheet. It should be emphasised that the original report does not itself use the terms “telocyte” or “telopode”: the equivalence between CMCs and telocytes is therefore an inference rather than a claim made by the authors, and must be treated with corresponding caution until tested directly. The proposal is nonetheless conceptually relevant, because it converges with earlier FIB-SEM tomographic reconstructions of telopodes, which had already indicated that at least some telopodes are flattened and ribbon-like rather than strictly cylindrical [44,45]. If fascial CMCs and canonical telocytes prove to be the same or overlapping populations, the apparently divergent “beaded thread” and “membranous sheet” descriptions could be reconciled as two-dimensional samplings of a single three-dimensional laminar stromal element. Resolving this will require direct ultrastructural and marker comparison of fascial CMCs and morphologically validated telocytes within the same study.

An additional stereological possibility should also be considered. The classical moniliform appearance of telopodes in two-dimensional ultrathin sections may not always correspond to a truly cylindrical “beaded” process in three dimensions. Instead, at least in some anatomical contexts, alternating thin podomeres and dilated podoms could partly reflect the plane and thickness of sectioning through an extremely thin, flattened, membrane-like cytoplasmic extension. In this interpretation, apparent beads or dilations in 2D may represent regions where the section intersects a broader laminar cytoplasmic sheet, organelle-rich domains, or folded membrane-like expansions, rather than discrete cylindrical swellings along a thread-like process. This alternative does not invalidate the classical telocyte description, but it cautions against assuming that every moniliform profile seen in 2D corresponds directly to a cylindrical telopode in 3D. Resolving this issue will require correlative approaches combining serial ultrathin sections, FIB-SEM or serial block-face SEM, spatial marker validation, and stereological modelling within the same tissue context.

The most serious conceptual challenge now comes from modern fibroblast biology. Modern lineage-tracing and single-cell atlas studies have reshaped the way stromal heterogeneity is interpreted. In skin, lineage-based work has identified dermal fibroblast populations with intrinsic fibrogenic potential, illustrating that fibrotic behaviour may be encoded in specific fibroblast lineages rather than in a single uniform stromal compartment [80]. More broadly, large-scale single-cell atlas initiatives, including Tabula Muris and the Human Cell Atlas, have shown that stromal cells form highly heterogeneous, tissue-specific transcriptional landscapes, with multiple fibroblast-like and mesenchymal subsets distributed across anatomical niches [81,82]. In this context, telocyte identity cannot be resolved by morphology or CD34/PDGFRα expression alone: it requires integration of ultrastructure, spatial context, molecular profiling, and functional testing. Single-cell and spatial approaches have revealed extensive stromal heterogeneity across tissues, including CD34-positive and PDGFRα-positive subsets in subepithelial, perivascular, adventitial, and deep stromal niches [34,36,61]. Against this background, the null hypothesis that telocytes may represent a specialised morphology or functional state within tissue-resident fibroblast-like populations has not yet been rigorously falsified. Falsification would require lineage tracing, fate mapping, spatial molecular profiling integrated with ultrastructure, and tissue-specific loss-of-function experiments. At present, only the intestinal Wnt-niche studies approach this level of functional evidence, and even there the relationship between molecularly defined Foxl1/Gli1 stromal cells and classical ultrastructural telocytes remains incompletely resolved [17,18,19,20].

A second unresolved issue is independent replication. Several foundational concepts, including the stromal synapse [31], miR-193 as a discriminating molecular signature [32], and the original three-class taxonomy of telocyte-derived extracellular vesicles [15,33], remain influential but incompletely re-examined with contemporary methods. These concepts should not be discarded, as each provided a productive hypothesis at an early stage of the field. However, they should be cited with calibrated language until independently reassessed using super-resolution microscopy, spatial proteomics, single-vesicle analysis, small-RNA sequencing, or comparable modern approaches.

We therefore advocate for constructive scepticism. Telocytes as an ultrastructural and spatial phenomenon are real: cells with extremely long, thin, moniliform processes and extensive stromal contacts have been repeatedly documented. Telocytes as a universally distinct cell lineage remain unproven. Telocyte functional necessity is robustly demonstrated only in the intestine, while most evidence outside the gut remains correlative or based on sufficiency. This position does not diminish the originality of the discovery era; rather, it clarifies what the next phase of the field must demonstrate. Independent replication, integration with mainstream stromal biology, molecular–spatial validation, and loss-of-function experiments outside the intestine will determine whether telocytes mature into a broadly accepted stromal cell category or are reinterpreted as a specialised network-forming phenotype within the fibroblast/stromal continuum.

The controversy is therefore not a crisis, but an opportunity. A field becomes stronger when it distinguishes what is established from what is plausible, and what is plausible from what remains speculative. The SNO framework and the two-dimensional evaluation matrix proposed in this review are offered in that spirit: not to defend all historical claims, but to provide tools for testing them.

## 11. Therapeutic Perspectives: Realistic Boundaries

In light of these evidential boundaries, therapeutic extrapolations must remain cautious. Telocytes are an attractive target for translational thinking because they organise stromal microenvironments and can be a source of regulatory paracrine signals. The translational literature is, however, dominated by sufficiency-based experiments—telocyte transplantation, conditioned medium, isolated extracellular vesicles—and by hypothesis-generating discussions [14,30,74]. Definitive clinical translation is not yet supported by the available evidence, and several technical questions remain open: standardisation of telocyte isolation, identity verification of the source cells, characterisation of vesicle cargo, dose standardisation, in vivo targeting, and long-term safety.

Within these limits, a small number of directions appear realistic. Preservation of endogenous telocyte networks—through avoidance or modulation of injuries that deplete or fragment them—is conceptually the most accessible strategy, although it presupposes a mechanistic understanding that is not yet available outside the gut. Telocyte-derived extracellular vesicles offer a tractable platform if production, cargo, and delivery can be standardised, with the principal evidence currently coming from endometrial regeneration models [30]. Organoid co-culture systems incorporating telocytes may improve the physiological relevance of in vitro models in the intestine, endometrium, skin, lung, and heart; the recent generation of immortalised intestinal telocyte lines [83] is a useful resource. We do not consider direct telocyte cell therapy to be realistic in the near term, given the difficulty of preserving telopode architecture through isolation and transplantation procedures and the absence of comparator data showing benefits over simpler alternatives. In oncology, the most defensible role for telocyte assessment is as a stromal biomarker rather than a therapeutic target, given the unresolved question of telocyte involvement in cancer-associated fibroblast biology.

## 12. Research Priorities for the Next Decade

The unresolved issues outlined above define a practical research agenda for the next decade. We propose a set of concrete priorities that are deliberately specific and, where possible, falsifiable. These priorities are summarised in Table 6 and then discussed in more detail below.

1. In vivo conditional ablation outside the intestine. The single most consequential gap in the field is the absence, outside the gut, of Porcn-deletion-equivalent experiments. Conditional ablation of Wnt secretion or of specific paracrine signals in skin, cardiac, and reproductive telocyte populations, with phenotypic readout in injury and homeostasis models, would directly test whether endogenous telocytes are necessary for tissue function in these organs. Lineage drivers will need to be identified or refined for each tissue.
2. Independent replication of single-laboratory foundational claims. Three foundational concepts in the field rest on single-laboratory descriptions and, to our knowledge, have not been independently re-examined with contemporary methods. We propose minimum experimental designs for each.
  (a) The stromal synapse [31]: Polarised localisation of presumptive pre- and post-synaptic markers (for example, scaffold proteins such as bassoon or piccolo on the telocyte side, complemented by E-cadherin–β-catenin clustering on the partner-cell side) by super-resolution STED or expansion microscopy in intact tissue, paired with immunogold TEM for ultrastructural confirmation, would provide independent evidence beyond the original TEM descriptions. Alternative hypotheses (non-polarised close apposition without dedicated molecular machinery) should explicitly be tested and reported.
  (b) The three-class extracellular vesicle taxonomy [33]: Single-vesicle nano-flow cytometry combined with immunogold labelling for tetraspanins (CD9, CD63, CD81), proteomic profiling of density-gradient-purified vesicles from freshly isolated telocytes, and complementary cryo-electron microscopy would either validate or revise the originally proposed 45 nm exosome/130 nm ectosome/multivesicular shed-vesicle scheme. Comparison with vesicles from neighbouring stromal populations (notably CD34-positive fibroblasts) should be included to establish whether the proposed taxonomy is telocyte-specific.
  (c) miR-193 as a discriminating signature [32]: In situ hybridisation for miR-193 across multiple telocyte-containing organs, combined with single-cell small-RNA sequencing of FACS-purified CD34-positive stromal cells and orthogonal fibroblast populations, would clarify its specificity for telocytes versus its expression in adjacent stromal populations. Without such validation across tissues, miR-193 should not be cited as a validated cell-type marker.

Either confirmation or revision of these three concepts would substantially advance the field; their continued unresolved status is a source of ambiguity that the next generation of experiments should address.

3. A high-resolution multi-organ ultrastructural atlas. Despite frequent assertions to the contrary, the field does not yet possess a comprehensive multi-organ atlas of telocyte ultrastructure obtained with modern correlative microscopy and standardised protocols. A coordinated effort using FIB-SEM tomography, serial block-face SEM, and electron tomography across at least the heart, lung, gut, skin, kidney, and reproductive organs, with quantitative network metrics, would provide the reference dataset that the field currently lacks.
4. Spatial molecular profiling integrated with ultrastructure. Spatial transcriptomics and multiplex imaging at high resolution, paired with ultrastructural validation in adjacent sections, would address the question of whether transcriptomic stromal clusters correspond to ultrastructurally defined telocytes. This integration is technically feasible with current methods and would resolve much of the present nomenclatural uncertainty.
5. Longitudinal studies and counter-cases. Cross-sectional comparison of healthy and diseased tissue is intrinsically limited for causal inference. Longitudinal animal models of fibrosis and chronic inflammation, with serial assessment of telocyte density, telopode integrity, and tissue outcome, would clarify whether telocyte changes precede, accompany, or follow disease progression. The field would also benefit from explicit reporting of cases that do not fit the network-failure expectation—telocyte preservation in fibrosis, fibrosis without telocyte alteration, and telocyte loss without fibrosis—that is currently underrepresented in the literature.
6. Standardised reporting. Adoption of a standard reporting framework—combining the two-dimensional identification/function matrix proposed here (Section 9) with explicit declaration of the methods used for each dimension—would allow more useful comparison across studies. Suggested terminology for future telocyte studies is provided in Supplementary Table S6. Reviews and meta-analyses currently struggle to integrate telocyte studies because the underlying methods vary in undocumented ways.

## 13. Conclusions

Two decades after the formal introduction of the term, telocytes are a recognisable ultrastructural and spatial stromal phenomenon. Their defining telopode architecture, their broad tissue distribution, and at least one specific functional role—Wnt-niche support in the intestine—are reasonably well established [13,14]. Other proposed functions, including antifibrotic regulation, immune calibration, reproductive endocrine integration, stromal boundary maintenance, and regenerative support, are biologically plausible and partially supported by sufficiency-based or correlative studies, but remain unverified by direct loss-of-function evidence outside the gut.

This review has argued that the field should now move beyond both uncritical acceptance and dismissive rejection. The appropriate position is constructive scepticism. Telocytes should not be reduced to CD34-positive spindle-shaped cells, but neither should every CD34-positive stromal cell with long processes be called a telocyte. Similarly, telocyte-derived secretome or extracellular vesicle effects should not be conflated with endogenous functional necessity. A more disciplined field requires explicit separation between identification confidence and functional confidence.

We have proposed two instruments for this next phase. The first is the Stromal Network Organiser framework, which consolidates overlapping models of telocyte function into a single testable structure centred on stromal network organisation and network-level dysfunction. The second is a two-dimensional evaluation matrix, which separates confidence in telocyte identification from confidence in telocyte function. Together, these tools allow the literature to be read more precisely: intestinal Wnt-niche studies occupy a high functional-confidence position; cardiac, dermal, and endometrial supplementation studies support sufficiency; disease-association studies remain largely correlative; and several foundational single-laboratory concepts require independent re-examination.

The most valuable contributions of the next decade are unlikely to come from further organ-mapping alone. They will come from independent replication of foundational claims, high-resolution multi-organ ultrastructural atlases, integration of spatial transcriptomics with ultrastructure, lineage tracing and fate mapping, and loss-of-function experiments outside the intestine. These studies will determine whether telocytes should be regarded as a distinct stromal cell type, a specialised network-forming phenotype, or a state within the broader fibroblast/stromal continuum. In that sense, the telocyte field is not closed; it is entering the phase in which its most important claims can finally be tested.

A key priority for the next decade will be the development of in situ and in vivo approaches that are capable of preserving telocyte architecture while simultaneously resolving their molecular identities and functional interactions within native tissues.

## Figures and Tables

**Table 1:** Twenty years of telocyte research: the conceptual evolution of the field. The table summarises the conceptual evolution of the field based on the literature reviewed in Section 1, Section 2 and Section 3 [1,2,3,4,5,6,7,8,13,14,15,16,44,45,46].

Era	Approximate Period	Main Concept	Representative Focus	Major Contribution	Main Limitation
Discovery/ICLC era	~2004–2010	Interstitial Cajal-like cells in extra-digestive organs	Myometrium, fallopian tube, myocardium, pancreas, mammary gland	Recognition of a previously overlooked stromal/interstitial cell population with long processes and strategic tissue localisation	Terminology still dependent on resemblance to interstitial cells of Cajal
Telocyte naming era	~2010 onward	Telocytes and telopodes	Ultrastructure, telopodes, podoms, podomeres	Establishment of a distinct conceptual and morphological identity	Functional roles largely inferred rather than directly demonstrated
Organ-mapping era	~2010–2020	Telocytes as widely distributed stromal cells	Heart, lung, skin, reproductive organs, digestive tract, urinary tract, synovium, pancreas, nervous system	Demonstrated broad anatomical distribution	Descriptive inflation; many studies relied on non-specific markers
Post-Popescu functional era	~2016 onward	Telocytes as functional stromal niche/network cells	Intestinal Wnt niche, extracellular vesicles, secretome, fibrosis, inflammation, regeneration	Shift from morphology to signalling, stem-cell niche biology, and disease mechanisms	Molecularly defined stromal populations do not always perfectly match classical ultrastructural telocytes
Network biology era	Emerging	Telocyte stromal network failure	Fibrosis, chronic inflammation, impaired regeneration, cancer stroma, reproductive disorders	Reframes telocytes as spatial organisers of tissue microenvironments	Requires integrated ultrastructural, spatial, molecular, and functional validation

**Table 2:** Telocytes versus major stromal and interstitial mimics. Marker and identity features are summarised from the literature reviewed in Section 1, Section 2 and Section 3 [5,34,35,36,43,59,60].

Feature	Telocytes	Fibroblasts	Myofibroblasts	Pericytes	Interstitial Cells of Cajal	Mesenchymal Stromal Cells
Main defining feature	Telopodes and stromal network connectivity	ECM production	Contractile matrix remodelling	Vascular mural localisation	GI pacemaker function	Multipotency/stromal support
Typical morphology	Small body, very long thin processes	Spindle-shaped, larger cytoplasm	Spindle-shaped, contractile stress fibres	Wrapped around capillaries	Branched interstitial cells	Variable, culture-dependent
Key markers	CD34, PDGFRα/β, vimentin, caveolin-1; tissue-dependent	Vimentin, collagen markers	αSMA, collagen I, fibronectin	NG2, PDGFRβ, desmin	c-kit, ANO1	CD73, CD90, CD105
Marker specificity	No single specific marker	No single specific marker	αSMA not exclusive	Perivascular context essential	c-kit/ANO1 more specific in GI	Culture phenotype may be artificial
Main function	Stromal connectivity, paracrine signalling, niche organisation	Matrix synthesis and maintenance	Wound contraction, fibrosis	Vessel stability	Pacemaking and neuromodulation	Regeneration/immunomodulation in culture
Main diagnostic risk	Overdiagnosis by CD34/PDGFRα alone	Confusion with telocytes	Confusion during fibrosis	Confusion near vessels	Historical confusion with ICLC	Confusion in culture studies

**Table 3:** Practical hardware and experimental to-do list for telocyte research. The table summarises methodological requirements for telocyte identification and functional validation, based on the literature on telocyte ultrastructure, telopodes, stromal mimics, three-dimensional reconstruction, marker limitations, extracellular vesicles, and functional perturbation studies [5,17,18,29,30,34,35,36,43,44,45,46,59,60,61].

Question	Required Hardware/Approach	What It Can Demonstrate	Main Limitation	Recommended Use
Is the cell compatible with a telocyte phenotype?	Transmission electron microscopy	Small cell body, very long thin telopodes, podomeres, podoms, caveolae, mitochondria, endoplasmic reticulum	Two-dimensional sections may misrepresent three-dimensional morphology	Minimal requirement for high-confidence morphology
Is the telopode truly a long-range three-dimensional structure?	Serial ultrathin sections, FIB-SEM, serial block-face SEM	Three-dimensional continuity, branching, laminar versus cylindrical geometry	Technically demanding; limited tissue volume	Needed to resolve stereological uncertainty
Is the cell stromal rather than haematologic or endothelial?	Immunohistochemistry/immunofluorescence with stromal, endothelial, immune, and pericyte markers	Supports exclusion of mimics	No single telocyte-specific marker exists	Must be combined with morphology
Is the cell distinct from fibroblasts?	Comparative marker panels, ultrastructure, spatial analysis, ECM-production assessment	Differences from fibroblasts, myofibroblasts, pericytes, and CD34-positive stromal cells	Partial marker overlap is expected	Essential in fibrotic and inflammatory tissues
Is the telocyte altered in disease?	Quantitative tissue analysis in defined disease contexts	Telocyte loss, fragmentation, altered density, network disruption, or stromal replacement	Association does not prove causality	Report disease context explicitly
Is the telocyte functionally required?	Conditional ablation, signalling deletion, rescue experiments, lineage tracing	Causal necessity for tissue function	Available mainly for intestinal Wnt niche	Required before claiming an indispensable function
Are secreted vesicles or mediators biologically active?	Vesicle isolation, conditioned medium, proteomics, RNA analysis, functional assays	Sufficiency of telocyte-derived material	Does not prove endogenous necessity	Interpret as biological activity, not proof of necessity
What remains to be done?	Correlative ultrastructure, spatial omics, perturbation, disease models	Integrated identity and function	Requires multi-method validation	Priority for the next decade

**Table 4:** Organ-specific telocyte evidence: proposed critical interpretation. The table summarises representative organ-specific telocyte evidence and provides a calibrated interpretation of the strength and limitations of the available literature. The references listed are representative rather than exhaustive; detailed discussions are provided in the corresponding sections of the main text.

Organ/System	Main Reported Localisation	Proposed Functions	Strength of Evidence	Main Limitation	Representative References
Myometrium	Between smooth muscle bundles, stromal compartments	Contractile coordination, hormone responsiveness, mechanical sensing	High historical relevance; important original evidence	Causality in uterine contractility remains incompletely proven	[2,46,64]
Fallopian tube	Tubal stroma, smooth muscle/epithelial interface	Tubal motility, gamete/embryo transport microenvironment, hormonal regulation	High historical relevance	Functional studies remain limited	[1,53,54,65]
Endometrium	Stromal and perivascular compartments	Cyclic repair, angiogenesis, immune regulation, fibrosis prevention	Emerging functional relevance	Human disease validation needed	[30]
Heart	Myocardial interstitium, near cardiomyocytes, vessels and progenitor-like cells	Paracrine support, repair, stromal coupling	Strong morphological tradition	Functional necessity still not fully established	[4,23,24,28,38,45]
Intestine	Subepithelial stromal niche	Wnt signalling, epithelial stem/progenitor support, niche organisation	Among the strongest post-Popescu functional evidence	Molecular niche cells must be integrated with ultrastructural telocyte criteria	[17,18,19,20,35,51]
Skin	Dermal stroma, near vessels, adnexa and fibroblasts	Wound repair, anti-fibrotic secretome, matrix regulation	Promising functional/secretome evidence	More in vivo validation needed	[21,22,29,44,52]
Lung	Interstitial and perivascular compartments	Repair, fibrosis modulation, immune–stromal signalling	Moderate	Many data remain descriptive	[16,50]
Synovium	Synovial stroma	Joint homeostasis, degenerative disease remodelling	Descriptive but relevant	Functional role unclear	[16]
Pancreas	Stromal/interstitial compartments	Tissue organisation, endocrine/exocrine microenvironment	Historical/descriptive relevance	Limited mechanistic data	[3]
Nervous system/peripheral nerves	Perineural or ganglionic stromal compartments	Mechanosensing, neural-stromal interaction	Emerging	Requires strict distinction from glial/perineural stromal cells	[56]
Cancer stroma	Tumour-associated stromal compartments	Stromal boundary regulation, CAF relationship, angiogenesis	Conceptually important	High risk of overinterpretation and marker confusion	[66,67]

**Table 5:** Two-dimensional evaluation matrix for telocyte studies. This matrix is proposed by the authors to separate identification confidence from functional confidence. It is based on the evidential distinctions discussed in Section 4 and Section 9.

Identification Confidence\Functional Confidence	F-0: Descriptive Only	F-1: Association	F-2: Sufficiency	F-3: Necessity
I-1: Low identification confidence Single marker only; no ultrastructural validation; mimics not excluded	Putative telocyte-like cells; descriptive claim only	Weak association; high risk of marker-based overinterpretation	Not recommended for functional claims unless source identity is independently validated	Not sufficient for necessity claims
I-2: Moderate identification confidence Marker-and-lineage or marker-panel evidence with morphological consistency; mimics considered	Credible telocyte-like population; suitable for mapping with caution	Moderate association when spatial context and disease correlation are provided	Acceptable evidence for sufficiency, e.g., transplantation, conditioned medium, EVs, co-culture, or organoids	Strong functional evidence if loss-of-function is rigorous, though ultrastructural correspondence should be discussed
I-3: High identification confidence Telopode ultrastructure documented by TEM/3D EM; spatial network integration; mimics excluded	High-confidence structural identification	Strong disease/topology association, but still not causal alone	Strong sufficiency evidence when structural identity and functional assays converge	Highest evidential standard: ultrastructural identity plus in vivo loss-of-function and rescue

**Table 6:** Future research agenda for the next decade. The priorities are proposed by the authors based on the evidence gaps identified throughout this review.

Priority	Key Question	Recommended Methods	Expected Impact
Integrated telocyte atlases	Which telocyte populations exist in each tissue?	TEM, 3D EM, multiplex IF, spatial transcriptomics, scRNA-seq	Defines tissue-specific telocyte identities
Lineage tracing	Are telocytes stable cells, dynamic states, or precursors of activated fibroblasts?	Fate mapping, inducible lineage models, trajectory analysis	Resolves telocyte–fibroblast–myofibroblast relationships
Functional perturbation	Are telocytes necessary for homeostasis or repair?	Ablation, knockdown, organoids, co-culture, rescue experiments	Moves field from association to causality
EV and secretome profiling	What do telocytes secrete and how does it change in disease?	Proteomics, miRNA profiling, lipidomics, EV characterisation	Enables telocyte-derived therapeutic strategies
Disease-stage mapping	When does telocyte disruption occur during disease?	Longitudinal models, human biopsies, spatial analysis	Distinguishes cause from consequence
Reproductive telocyte biology	How do hormones regulate telocyte networks?	Cycle-stage tissue analysis, hormone assays, endometrial organoids	Clarifies roles in fertility, endometriosis and repair
Fibrosis models	Do telocytes prevent or reverse myofibroblast dominance?	TGF-β models, anti-fibrotic assays, lineage tracing	Tests the telocyte–myofibroblast balance concept
Cancer stromal mapping	Are telocytes distinct from CAFs in tumours?	Spatial single-cell atlases, multiplex imaging, EM validation	Clarifies cancer relevance
Therapeutic development	Can telocyte functions be preserved or mimicked?	EV therapy, secretome, biomaterials, stromal niche engineering	Builds translational telocyte biology
Terminology standardisation	What should be called telocyte, telocyte-like, or stromal niche cell?	Consensus criteria, evidence grading	Prevents conceptual dilution

## Data Availability

No new data were generated for this review. All cited studies are referenced and publicly available through their respective journals.

## Supplementary Materials

The following supporting information can be downloaded at: https://www.mdpi.com/article/10.3390/ijms27146204/s1.
