# Supplementary material for: Telocytes Twenty Years on: A Critical Reappraisal of Identity, Function, and Pathological Relevance
Source: Int J Mol Sci. 2026 Jul 11;27(14):6204. doi: 10.3390/ijms27146204 (PMC13411473; doi:10.3390/ijms27146204)
Supplement: Supplementary file 1 [file ijms-27-06204-s001.zip › ijms-4339458-supplementary.pdf]

Supplementary Table S1. Minimal criteria proposed for telocyte identification

Criterion	Required or supportive?	Recommended methods	Rationale	Common pitfall
Telopodes	Required for high-confidence identification	TEM, serial block-face SEM, high-resolution confocal when appropriate	Telopodes are the defining morphological feature	Calling any long process a telopode
Small cell body with long, thin processes	Supportive	TEM, confocal microscopy, 3D imaging	Helps distinguish telocytes from fibroblasts and other stromal cells	Overinterpretation in 2D sections
Podoms and podomeres	Strongly supportive	TEM / 3D EM	Characteristic moniliform organization of telopodes	Often missed without ultrastructure
Interstitial/stromal localization	Required	Histology, spatial imaging, multiplex IF	Telocytes are spatially defined stromal/interstitial cells	Ignoring tissue context
Marker panel	Supportive, not diagnostic	CD34, PDGFRα/β, vimentin, c-kit, caveolin-1, tissue-specific markers	Helps phenotype the population	Treating CD34 or PDGFRα as diagnostic
Exclusion of mimics	Required	Multiplex IF, EM, anatomical localization	Distinguishes telocytes from fibroblasts, pericytes, endothelial cells, ICC, MSCs	Assuming all CD34+ stromal cells are telocytes
Network formation	Strongly supportive	3D imaging, EM, spatial analysis	Supports the stromal network-cell concept	Evaluating cells in isolation
Functional evidence	Required for mechanistic claims	Co-culture, organoids, ablation, lineage tracing, EV/secretome assays	Establishes biological relevance	Inferring function from proximity alone

Supplementary Table S2. Methodological pitfalls in telocyte research

Pitfall	Why it is problematic	Consequence	Recommended correction
Equating CD34 positivity with telocyte identity	CD34 is expressed by multiple stromal and vascular-associated cells	Overdiagnosis of telocytes	Use CD34 only as part of a broader panel and spatial/morphological analysis
Using PDGFRα as a standalone marker	PDGFRα labels broad mesenchymal compartments	Confusion with fibroblast-like niche cells	Combine with morphology, tissue context, and functional data
Ignoring ultrastructure	Telopodes may not be reliably identified by routine light microscopy	Weak cellular identification	Use TEM/3D EM in foundational or controversial claims
Inferring function from location	Proximity does not prove regulation	Overstated claims about stem cells, immune cells, or fibrosis	Use perturbation, co-culture, organoids, or disease models
Confusing telocytes with fibroblasts or MSCs	Shared markers and culture-induced morphology overlap	Conceptual dilution	Define exclusion criteria and use precise terminology
Overgeneralizing animal data to humans	Telocyte phenotype may be species-specific	Weak translational claims	Validate in human tissues
Treating telocytes as universally beneficial	Telocyte function may be context-dependent	Oversimplified disease models	Consider protective, permissive, or maladaptive roles
Publishing organ-mapping without mechanism	Presence alone no longer advances the field	Descriptive inflation	Link new organ reports to function, disease, or spatial biology

Supplementary Table S3. High-confidence versus weak telocyte claims

Claim type	Strong formulation	Weak / problematic formulation
Identity	Cells with ultrastructurally confirmed telopodes and supportive marker profile were identified.	CD34-positive cells were considered telocytes.
Function	Telocyte-derived signals altered fibroblast activation in functional assays.	Telocytes were near fibroblasts, suggesting they regulate fibrosis.
Disease relevance	Telocyte network disruption correlated with disease stage and functional markers of remodelling.	Telocytes were reduced in disease, therefore they cause disease.
Stem-cell niche	Telocyte-like stromal cells provided niche signals required for epithelial progenitor maintenance.	Telocytes are close to stem cells, therefore they support stem cells.
Inflammation	Telocytes modulated macrophage phenotype in an experimental model.	Telocytes are anti-inflammatory cells.
Cancer	Telocyte-like stromal networks may be altered in tumor microenvironments.	Telocytes prevent or promote cancer.
Therapy	Telocyte-derived EVs may represent a candidate paracrine strategy requiring validation.	Telocytes can be used as treatment for fibrosis.

Supplementary Table S4. Proposed models emerging from twenty years of telocyte research

Proposed model	Central idea	Mechanistic components	Disease relevance	Testable predictions	Representative references
Telocyte Stromal Network Failure Model	Disease involves disruption of telocyte-mediated stromal connectivity	Telocyte depletion, telopode fragmentation, network uncoupling, altered EVs	Fibrosis, inflammation, failed regeneration, cancer stroma	Network disruption should correlate with disease severity better than cell number alone	[11, 18, 19, 20, 21, 23, 32, 67, 68]
Telocyte-associated stromal network dysfunction 2.0	Telocyte-associated stromal network dysfunction is a network-level disorder, not merely telocyte loss	Loss of contacts, altered secretome, niche failure, immune dysregulation	Chronic tissue remodelling disorders	Tissues may show telocyte-associated stromal network dysfunction despite preserved telocyte counts	[20, 21, 23, 67, 68]
Telocyte Paracrine Shield Model	Telocytes maintain stromal quiescence through EVs and soluble mediators	EV cargo, anti-fibrotic signals, fibroblast restraint	Fibrosis, scarring, chronic inflammation	Loss of telocyte secretome should increase myofibroblast activation	[22,36,51,64]
Telocyte–Myofibroblast Balance Model	Repair depends on balance between telocyte networks and myofibroblast activation	TGF-β, αSMA, ECM deposition, telopode loss	Fibrotic diseases	Successful repair should restore telocyte networks after transient myofibroblast activation	[20,21,22,34]
Reproductive Telocyte Endocrine-Niche Model	Reproductive telocytes integrate hormonal, contractile, vascular, and regenerative signals	Estrogen/progesterone response, smooth muscle communication, angiogenesis, endometrial repair	Infertility, endometriosis, adhesions, uterine dysfunction	Telocyte phenotype should vary across cycle, pregnancy, and reproductive disease	[1,2,19,27,64,70]
Telocyte Immune Rheostat Model	Telocytes tune the intensity and spatial organization of inflammation	Macrophage/mast-cell interactions, cytokines, EVs, stromal neighborhoods	Chronic inflammation, autoimmune-like remodelling, fibrosis	Telocyte disruption should alter immune-cell localization and resolution	[65,66]

Telocyte Stromal Boundary Model	Telocytes maintain stromal boundaries between epithelial, vascular, immune, and ECM compartments	Telopode networks, epithelial-stromal interface, vascular contacts	Cancer, invasion, CAF remodelling	Tumors should show loss or corruption of telocyte-defined stromal boundaries	[57,59]

Supplementary Table S5. Telocytes in disease: proposed mechanisms and evidence caution

Disease context	Proposed telocyte alteration	Possible mechanism	Interpretation strength	Caution	Representative references
Fibrosis	Telocyte loss, telopode fragmentation, network collapse	Loss of paracrine restraint, myofibroblast dominance, ECM accumulation	Conceptually strong	Causality often not proven	[20,21,22,33,58]
Chronic inflammation	Altered telocyte-immune interactions	Immune rheostat failure, macrophage/mast-cell dysregulation	Moderate/emerging	Context-dependent; not always anti-inflammatory	[46,47,65,66]
Impaired regeneration	Loss of niche support and stromal coordination	Reduced Wnt/EV/paracrine signalling, poor progenitor support	Strongest in intestine; emerging elsewhere	Organ-specific mechanisms differ	[13,14,15,38,64]
Cancer stroma	Telocyte depletion, boundary failure or CAF overlap	Stromal disorganization, altered angiogenesis, immune remodelling	Conceptually important	High risk of confusion with CAFs/CD34+ stroma	[57,59]
Endometriosis	Altered reproductive telocyte immune/angiogenic signalling	Macrophage modulation, VEGF/MMP/NF-κB pathways	Promising experimental evidence	Human validation needed	[64,65,66]
Intrauterine adhesions / endometrial fibrosis	Telocyte network loss or EV dysfunction	Failed endometrial repair, fibrosis, impaired angiogenesis	Emerging	Needs rigorous human studies	[64]
Cardiac injury	Telocyte depletion or paracrine dysfunction	Reduced repair support, altered cardiomyocyte/stromal communication	Moderate	Functional rescue data needed	[40,41,51]
Skin scarring	Loss of telocyte anti-fibrotic secretome	Fibroblast-to-myofibroblast transition	Promising	More in vivo evidence required	[20,21,22,52,58]

Supplementary Table S6. Proposed terminology for future telocyte studies

Term	Recommended use	Minimum evidence	Avoid using when
Telocyte	For cells with convincing telopodes and stromal network localization	Ultrastructure or very strong spatial/morphological validation plus supportive markers	Only CD34/PDGFRα positivity is shown
Telocyte-like cell	For cells resembling telocytes but lacking complete validation	Suggestive morphology and marker profile	Functional claims are strong but identity is weak
Telocyte network	For spatially connected telocytes with intercellular relationships	3D/spatial evidence of connectivity	Cells are analyzed only after dissociation
Telocyte-associated stromal network dysfunction	For disease states with telocyte network disruption	Evidence of telocyte loss, telopode damage, or functional impairment	Telocytes are merely present in diseased tissue

Telocyte-derived EVs	For vesicles isolated from rigorously defined telocytes	Validated cell source and EV characterization	Cell culture contains mixed stromal populations
Stromal niche cell	For molecularly defined stromal cells with niche function	Functional niche evidence	Telopodes are not assessed
CD34+ stromal cell	For CD34-positive interstitial cells without telopode validation	CD34 staining and stromal localization	The authors intend to imply telocyte identity without proof
